# Dengue and Chikungunya virus co-infection in major metropolitan cities of provinces of Punjab and Khyber Pakhtunkhwa: A multi-center study

**DOI:** 10.1371/journal.pntd.0009802

**Published:** 2021-09-23

**Authors:** Faiz Ahmed Raza, Hasnain Javed, Muhammad Mujeeb Khan, Obaid Ullah, Areeba Fatima, Muhammad Zaheer, Saima Mohsin, Shahida Hasnain, Ruqyya Khalid, Arslan Ahmed Salam

**Affiliations:** 1 Institute of Microbiology and Molecular Genetics, University of the Punjab, Lahore, Pakistan; 2 National Institute of Health (NIH), Health Research Institute, Research Centre, Fatima Jinnah Medical University, Lahore, Pakistan; 3 Advance Diagnostic Laboratory, Punjab AIDS Control Program, Lahore, Pakistan; 4 Rawalpindi Medical University, Rawalpindi, Pakistan; 5 National Institute of Health (NIH), Health Research Institute, Research Centre, Khyber Medical College, Peshawar, Pakistan; 6 Department of Biochemistry, Kinnaird College for Women, Lahore, Pakistan; 7 National Institute of Health (NIH), Health Research Institute, Research Centre, Shaikh Zayed Hospital, Lahore, Pakistan; 8 National Institute of Health (NIH), Health Research Institute, Head Office, Islamabad, Pakistan; University of Heidelberg, GERMANY

## Abstract

Dengue has become endemic in Pakistan with annual recurrence. A sudden increase in the dengue cases was reported from Rawalpindi in 2016, while an outbreak occurred for the first time in Peshawar in 2017. Therefore, a multi-center study was carried out to determine the circulating dengue virus (DENV) serotypes and Chikungunya virus (CHIKV) co-infection in Lahore, Rawalpindi, and Peshawar cities in 2016–18. A hospital-based cross-sectional study was carried out in Lahore and Rawalpindi in 2016–18, while a community-based study was carried out in Peshawar in 2017. The study participants were tested for dengue NS1 antigen using an immunochromatographic device while anti-dengue IgM/IgG antibodies were detected by indirect ELISA. All NS1 positive samples were used for DENV serotyping using multiplex real-time PCR assay. Additionally, dengue samples were tested for CHIKV co-infection using IgM/IgG ELISA. A total of 6291 samples were collected among which 8.11% were NS1 positive while 2.5% were PCR positive. DENV-2 was the most common serotype (75.5%) detected, followed by DENV-1 in 16.1%, DENV-3 in 3.9% and DENV-4 in 0.7% while DENV-1 and DENV-4 concurrent infections were detected in 3.9% samples. DENV-1 was the predominant serotype (62.5%) detected from Lahore and Rawalpindi, while DENV-2 was the only serotype detected from Peshawar. Comorbidities resulted in a significant increase (p-value<0.001) in the duration of hospital stay of the patients. Type 2 diabetes mellitus substantially (p-value = 0.004) contributed to the severity of the disease. Among a total of 590 dengue positive samples, 11.8% were also positive for CHIKV co-infection. Co-circulation of multiple DENV serotypes and CHIKV infection in Pakistan is a worrisome situation demanding the urgent attention of the public health experts to strengthen vector surveillance.

## Author summary

Mosquitoes are responsible for the transmission of many different types of infectious pathogens and parasites to humans. Some of these pathogens include viruses like dengue and chikungunya viruses which cause diseases in humans. Dengue virus has four different types (1, 2, 3, and 4) present in nature. In this study, we detected all four dengue virus types in hospitalized patients in Lahore and Rawalpindi. While only dengue virus type-2 was detected from Peshawar city. The secondary infections with different dengue virus types than the previous one might result in a more severe form of the disease. Therefore, the population of Peshawar is at risk of developing a severe form of dengue fever in the future in case of infection from another dengue virus type. The data of hospitalized patients showed that other diseases in addition to dengue fever result in an increased hospital stay. Comorbidities like diabetes also increase the risk of developing a more severe form of dengue fever. Combined infections of both dengue and chikungunya viruses were detected from Lahore, Rawalpindi, and Peshawar. The transmission of dengue and chikungunya viruses and the co-circulation of multiple dengue virus types not only impose public health burden but also result in economic losses.

## Introduction

Dengue is caused by a positive-sense single-stranded RNA virus known as dengue virus (DENV) belonging to the family Flaviviridae genus *Flavivirus*. The virus has four antigenically distinct serotypes (DENV-1 to 4) [1], which are maintained in a human-to-mosquito-to-human transmission cycle [2]. In addition to these previously known four serotypes, a fifth serotype, DENV-5, circulating in the macaques has also been proposed from Malaysia in 2013 [3]. Infection with one of the serotypes imparts lifelong immunity against that particular serotype and temporary partial immunity against the other serotypes. Secondary infection with the different serotypes increases the risk of severe dengue hemorrhagic fever [4].

The DENV is transmitted to humans mainly by female *Aedes (Stegomyia) aegypti* (*Ae*. *aegypti*) and to a lesser extent by *Ae*. *albopictus* mosquitoes [1]. *Ae*. *aegypti* is also a vector for Zika, Chikungunya, and Yellow Fever viruses [5]. Previously dengue-free countries like Pakistan, Saudi Arabia, Yemen, Sudan, and Madagascar are now facing outbreaks of dengue infection. Because of the subtropical location of Pakistan, it is the hotspot for many vector-borne diseases like dengue, malaria, leishmaniasis, West Nile virus, and Crimean-Congo hemorrhagic fever, etc. [6]. Dengue vector exists in the Indian subcontinent even before its partition into Pakistan and India in 1947 [7]. Later, the *Ae*. *aegypti* mosquito was reported from various cities of provinces of Punjab (Attock and Lahore), Khyber Pakhtunkhwa (Peshawar and Dera Ismail Khan), and Sindh (Karachi and Larkana) in 1950 [8]. Similarly, *Ae*. *albopictus* mosquitoes were collected from Changa Manga National Forest near Lahore in 1964 [9]. Therefore, epidemic potential already exists in the region, however, no case of dengue fever was documented till 1968 when 14 children (1–10 years old) were reported with encephalitis from Lahore. The virus was identified as DENV-3 from the cerebrospinal fluid of a patient [8]. Later, in 1978 cross-reacting antibodies against DENV-3 were detected in the serum samples collected from Changa Manga National Forest [10]. Similarly, serosurveys carried out in 1983/1985 in Karachi detected antibodies against DENV-2 in the healthy population [11].

The first major outbreak of dengue hemorrhagic fever (DHF) due to DENV-1 and DENV-2 affecting thousands of people was reported from Karachi in 1994 [12]. Another outbreak due to DENV-1 and DENV -2 was reported from Baluchistan in 1995 [13]. After a decade, a major outbreak of DHF due to DENV-3 in the population previously sensitized to DENV-1 and DENV-2 was reported from Karachi in 2005 [14]. The largest outbreak with more than 3640 cases of dengue fever was reported in Karachi in 2006 due to DENV-2 and DENV-3 [15,16]. Although dengue cases were detected initially from Lahore, later the disease remained limited to the port city of Karachi, Sindh province with sporadic cases from other cities. However, no major outbreak was reported from other provinces until 2008, when 1407 cases of dengue fever were reported from Lahore, Punjab due to DENV-2 (subtype IV), DENV-3 (subtype III), and DENV-4 [17,18]. The disease spread to the neighboring cities of Lahore by 2010 resulting in outbreaks due to all four DENV serotypes in Sheikhupura and Gujranwala in addition to Lahore [19].

Punjab province was hit by the largest outbreak of dengue fever in the history of Pakistan affecting several thousands of people in Lahore and Faisalabad as the epicenters in 2011. After 2011–12, another dengue outbreak for the first time in the history of Pakistan was reported from Swat district, Khyber Pakhtunkhwa in 2013 [20,21]. The outbreak spread to the other districts of the province in 2014–15 and small-scale outbreaks were reported from Mansehra, Kohat, and Malakand areas, Khyber Pakhtunkhwa [20]. The frequency of dengue patients suddenly increased in two other districts including Rawalpindi and Peshawar in 2017–18, therefore, a multicenter study was carried out in major districts of Khyber Pakhtunkhwa (Peshawar) and Punjab (Lahore and Rawalpindi) to determine the circulating serotypes in these cities. Further, these samples collected from all three districts were also tested for the Chikungunya virus (CHIKV) co-infection.

## Materials and methods

### Ethical considerations

Ethical clearance for the research study was taken from the Ethical Review Committee (ERC), Fatima Jinnah Medical University, Lahore-Pakistan (No.26-PHRC/IERB). Written informed consent was obtained from all the study participants or the legal guardians in the case of children (<18 years old).

### Study sites, sampling, and data collection

**Fig 1:**
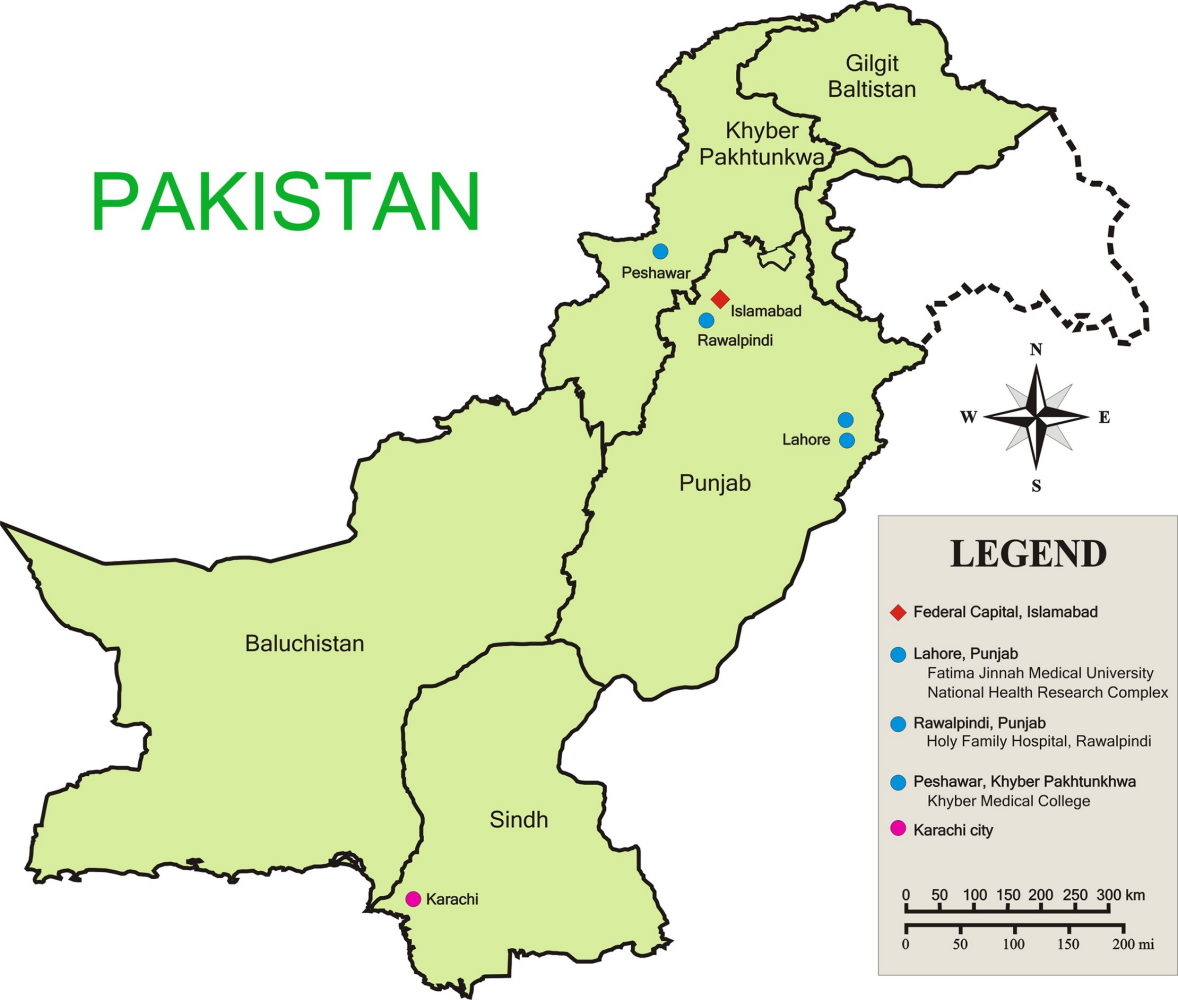
Map of Pakistan showing study sites in provinces of Punjab and Khyber Pakhtunkhwa. The location of Karachi city is also shown where the first dengue outbreak was reported in Pakistan. The map was prepared in CorelDraw software. Pakistan Base Map URL: https://commons.wikimedia.org/wiki/File:Pakistan_Base_Map.png.

A hospital-based cross-sectional study was carried out in the province of Punjab at Sir Ganga Ram Hospital, Lahore; Shaikh Zayed Hospital, Lahore; and Holy Family Hospital, Rawalpindi during 2016–18. Lahore is the largest city of the province Punjab and the second-largest city of Pakistan with a population of 11.3 million [22] located between latitude 31.5204° N and longitude 74.3587° E. The city has a semi-arid climate with five seasons. The temperature in summer reaches as high as 50°C to as low as -1°C in winter. Conditions become especially conducive for the proliferation of mosquitoes in the post-monsoon period in July-August each year. Fig 1 shows the geographical locations of the study sites.

Rawalpindi is the fourth largest city in Pakistan with a population of about 2.1 million located between latitude 33.5651° N, and longitude 73.0169° E. The city is located near the federal capital Islamabad near the foothills of Himalaya’s mountain range. The city has a humid tropical climate favoring the proliferation of the DENV, especially after monsoon rainfall each year (Fig 1).

Revised criteria of Dengue Expert Advisory Group (DEAG) [23] were adopted for the recruitment of hospitalized DF cases. All the patients were considered as probable cases of DF if presented with fever from 2–10 days and having two or more of the following symptoms: retro-orbital pain, headache, rash, myalgia, arthralgia/severe backache/bone pains, rash, hemorrhagic manifestations, abdominal pain, reduced urinary output, irritability in infants, thrombocytopenia (≤100,000 cells/mm^3^) and leukopenia (<4000 cells/mm^3^). The DENV infection was confirmed using laboratory tests (i.e. NS1 antigen or IgM or PCR positive). DEAG criteria were part of the initial workup by the tertiary care hospitals and only confirmed DF patients were hospitalized for management while those negative for lab tests were usually managed on an outdoor basis.

Confirmed dengue patients were classified according to disease severity into less severe dengue fever (DF) and more severe dengue hemorrhagic fever (DHF) using World Health Organization (WHO) case definition [24]. Briefly, a patient was labeled as a case of DHF if he/she had (1) fever for 2–7 days; (2) one or more hemorrhagic manifestations (positive tourniquet test, petechiae, ecchymosis or purpura, bleeding from the mucosa, injection sites, and hematemesis and/or melena); (3) thrombocytopenia (platelet count less than 100,000/mm^3^); and (4) evidence of plasma leakage (increase of ≥20% hematocrit (HCT) from the baseline of patient or population of the same age) [24].

During the study period, a large-scale dengue outbreak occurred in Peshawar city in 2017 leading to 24,807 laboratory-confirmed cases and 69 deaths [25]. Therefore, a community-based cross-sectional study was also carried out to determine the circulating DENV serotypes in Peshawar. Peshawar is the sixth-largest metropolitan city of Pakistan and the provincial capital of Khyber Pakhtunkhwa. It is located in the non-monsoon region and has a hot semi-arid climate, however, the city receives rainfall both in summer and winter especially between February and April. The temperature in summer surpasses 40°C and it declines to as low as 4°C in the winter (Fig 1). A total of 6000 participants were enrolled in a community-wide study carried out in Town 3 and 4 (union councils # 37, 38, 39, 40, 57, 59, 60, 79, 81, 87, and 91) of the Peshawar city during Sep-Nov, 2017. The residents were invited to participate in this study through a DENV infection screening and awareness campaign carried out by the Pakistan Red Crescent Society in collaboration with Pakistan Health Research Council. The participants were recruited irrespective of their age and gender. However, people with fever and having two or more dengue-like symptoms were excluded from the study, and they were referred to specialized treatment centers for proper diagnosis and management of the disease. Demographic details including name, age, and gender were obtained from each participant and blood samples were drawn according to standard protocol.

### Immunodiagnosis of dengue virus infection

The serum samples obtained from the participants were tested through immunochromatographic (ICT) TruQuick DENG IgG/IgM/NS1 Rapid Test-Cassette (Meridian Biosciences USA; Catalog # TQ2025) for the qualitative detection of dengue NS1 antigen as per manufacturer’s instructions. The kit had a high sensitivity of 95.8% and a specificity of 96.1%. Dengue immunoglobulin M (IgM) and G (IgG) antibodies were measured by indirect IgM/IgG ELISA, using commercially available kits (Human GmbH, Germany; Catalog # 51140 and 51240 respectively). The manufacturer reported the sensitivity of 96.3% and specificity of 91.3% for the IgM ELISA kit (http://www.human.de/data/gb/vr/el-denm.pdf) while the sensitivity of 89.3% and specificity of 94.1% for IgG ELISA kit (http://www.human-de.com/data/gb/vr/el-deng.pdf). Absorbance was measured at 450nm using a microtiter plate reader (HumaReader, Human GmbH, Germany). Patients having an index value less than the cutoff were considered as negative for dengue infection. Two different matrixes were followed for hospital-based and community-based studies. Hospitalized patients (Rawalpindi and Lahore) were tested for both NS1 antigen and IgM/IgG antibodies. While participants recruited from the community (Peshawar) were tested only for NS1 antigen.

### Multiplex real-time PCR (qPCR) assay

Serum samples of the confirmed dengue cases (i.e. positive for NS1 antigen) were used for serotype analysis. DENV RNA was extracted from serum samples using a commercially available viral RNA extraction kit (Vivantis, Malaysia; Catalog # GF-RD-300). DENV serotype analysis was carried out using multiplex real-time abTES DEN4 qPCR I kit (AITbiotech Singapore; Catalog # 300156) for the detection of all four serotypes. The kit had high sensitivity (92 to 100%) and specificity (96 to 100%) for all DENV serotypes [26,27]. An internal control (IC) was used to check the possible PCR inhibition. The kit was used with Bio-Rad CFX96 PCR platform which allowed multiplexing up to 5 targets with minimum sample volumes (as low as 10μl). Viral serotypes were identified using five excitation channels for each of four DENV serotypes and IC.

### Diagnosis of Chikungunya virus co-infection

Serum samples collected from dengue patients were also tested for Chikungunya virus (CHIKV) co-infection through ELISA. IgM/IgG antibodies targeting CHIKV envelope glycoproteins E2/E1 in the patient serum were detected using CHIKjj *Detect* enzyme-linked sandwich-type immunoassay kit (InBios International, Inc., USA). The kits were developed using innovative technology [28]. The IgM kit had a high sensitivity (100%) and specificity (100%) [29], while the IgG kit had >90% sensitivity and specificity. The kit had a high analytical specificity and showed no/minimal cross-reactivity against commonly known viral and other disease agents especially against DENV (100%), Japanese encephalitis virus (100%), West Nile virus (100%), hepatitis C virus (100%) and human immunodeficiency virus (100%). Immune status ratio (ISR) i.e. test sample OD ÷ cut-off control OD) of ≥1.0 was considered as “reactive” while <1.0 was considered as “non-reactive”. Any samples in the range of 0.9 to 1.10 ISR were retested in duplicates for confirmation of the results.

### Statistical analysis

Statistical Package for the Social Sciences (SPSS) version 19 was used for data entry and analysis. The non-parametric Mann-Whitney U test was used to calculate the p-value for age; and the χ^2^ test, for other categorical variables. A significance level of 5% was used for analysis. A 2-tailed p-value ≤0.05 was considered statistically significant.

## Results

### Circulating DENV serotypes in Lahore and Rawalpindi

All the confirmed dengue patients (*n =* 291) reported in 2016–18 to the hospitals in Lahore (*n* = 81), and Rawalpindi (*n* = 210) were recruited in this study. Study participants included 222 (76.3%) males and 69 (23.7%) females. The median age of the patients was 30 years (range = 7 to 78 years). There were 28 (9.6%) children (till 16 years) and 263 (90.4%) adults. There were 133 (45.7%) young adults (17 to 30 years old) among adult patients. The highest prevalence (46%) of dengue patients was observed between 16 to 30 years followed by 29.9% in the age group of 31 to 45 years. A similar trend was noted in the age distributions among different study populations from various geographical locations (Table 1).

**Table 1:** Age distribution of the participants from different study sites.

Age groups (years)	Total *n* (%)	Hospital-based studies				Community-based study*	
		Lahore *n* (%)		Rawalpindi *n* (%)		Peshawar *n* (%)	
		Male	Female	Male	Female	Male	Female
≤15	104 (17.6)	7 (8.6)	3 (3.7)	14 (6.7)	3 (1.4)	28 (9.4)	49 (16.4)
16–30	277 (47)	26 (32.1)	14 (17.3)	77 (36.7)	17 (8.1)	69 (23.1)	74 (24.7)
31–45	143 (24.2)	15 (18.5)	9 (11.1)	51 (24.3)	12 (5.7)	29 (9.7)	27 (9.0)
46–60	47 (8)	2 (2.5)	2 (2.5)	20 (9.5)	5 (2.4)	9 (3.0)	9 (3.0)
≥60	19 (3.2)	2 (2.5)	1 (1.2)	8 (3.8)	3 (1.4)	3 (1.0)	2 (0.7)

*Data was stratified into different age groups for the positive cases only

Headache was the most common symptom reported in 242 (83.6%) participants followed by myalgia/arthralgia in 129 (44.3%), abdominal pain in 126 (43.3%), vomiting in 118 (40.5%), cough in 102 (35.1%), cold/clammy skin in 93 (32%), sore throat in 87 (29.9%), retro-orbital pain in 77 (26.5%), diarrhea in 70 (24.1%) rash in 68 (23.4%), restlessness in 67 (23%), anorexia in 40 (13.7%) and periorbital puffiness in 5 (1.7%) participants. Among 291 patients, 59 different types of hemorrhages were reported including petechiae in 17 (5.8%) participants followed by hematemesis in 11 (3.8%), epistaxis in 8 (2.7%), gingivitis in 7 (2.4%), melena in 5 (1.7%), hematuria in 5 (1.7%), ecchymosis in 4 (1.4%), hematochezia in 2 (0.7%) and purpura in 2 (0.7%) participants. Spleen and liver were enlarged in 22 (7.6%) and 24 (8.3%) patients respectively, while abdominal tenderness was reported in 3 (1%) patients. The patients were reported with an average of 6.4 days of fever while they stayed at the hospital for 3.2 days for management (Table 2).

**Table 2:** Clinico-epidemiological features of hospitalized patients reported with DENV infection alone or with comorbidities or with DENV-CHIKV co-infection in Lahore and Rawalpindi cities.

Characteristics	Total *n* (%)	Severity of DF		p-value*	Comorbidities		p-value*	DENV-CHIKV coinfection		p-value*
		DF *n* (%)	DHF *n* (%)		DENV infection *n* (%)	Comorbidities *n* (%)		DENV infection *n* (%)	DENV-CHIKV coinfection *n* (%)	
Age [median (interquartile range), years]	30 (22–40)	30 (22–40)	33 (28–40)	0.298	29 (20–36)	33 (25–45)	0.005	30 (22–40)	30 (18–40)	0.594
**Gender**										
Male	222 (76.3)	190 (76.9)	32 (72.7)	0.566	147 (77.4)	75 (74.3)	0.565	180 (75.9)	42 (77.8)	0.593
Female	69 (23.7)	57 (23.1)	12 (27.3)		43 (22.6)	26 (25.7)		57 (24.1)	12 (22.2)	
**Clinical Presentation**										
Headache	242 (83.6)	209 (84.6)	33 (75)	0.092	147 (77.4)	95 (94.1)	<0.001	199 (84.0)	43 (79.6)	0.279
Myalgia/ arthralgia	129 (44.3)	101 (40.8)	28 (63.6)	0.004	105 (55.3)	24 (23.8)	<0.001	107 (45.1)	22 (40.7)	0.332
Vomiting	118 (40.5)	98 (39.7)	20 (45.5)	0.289	54 (28.4)	64 (63.4)	<0.001	99 (41.8)	19 (35.2)	0.232
Abdominal pain	126 (43.3)	107 (43.3)	19 (43.2)	0.561	70 (36.8)	56 (55.4)	0.003	103 (43.5)	23 (42.6)	0.516
Rash	68 (23.4)	48 (19.4)	20 (45.5)	<0.001	46 (24.2)	22 (21.8)	0.666	53 (22.4)	15 (27.8)	0.248
Diarrhea	70 (24.1)	67 (27.1)	3 (6.8)	0.002	24 (12.6)	46 (45.5)	<0.001	56 (23.6)	14 (25.9)	0.421
Anorexia	40 (13.7)	28 (11.3)	12 (27.3)	0.007	30 (15.8)	10 (9.9)	0.211	35 (14.8)	5 (9.3)	0.203
Sore throat	87 (29.9)	80 (32.3)	7 (15.9)	0.018	27 (14.2)	60 (59.4)	<0.001	72 (30.4)	15 (27.8)	0.421
Retro-orbital pain	77 (26.5)	62 (25.1)	15 (34.1)	0.145	56 (29.5)	21 (20.8)	0.126	57 (24.1)	20 (37.0)	0.040
Cough	102 (35.1)	89 (36)	13 (29.5)	0.257	39 (20.5)	63 (62.4)	<0.001	84 (35.4)	18 (33.3)	0.450
Cold/clammy skin	93 (32.0)	84 (34)	9 (20.5)	0.052	28 (14.7)	65 (64.4)	<0.001	75 (31.6)	16 (29.6)	0.455
Restlessness	67 (23.0)	61 (24.7)	6 (13.6)	0.075	27 (14.2)	40 (39.6)	<0.001	49 (20.7)	18 (33.3)	0.038
Periorbital puffiness	5 (1.7)	3 (1.2)	2 (4.6)	0.166	3 (1.6)	2 (2.0)	0.802	4 (1.7)	1 (1.9)	0.645
**Hemorrhages**										
Epistaxis (nose bleed)	8 (2.7)	6 (2.4)	2 (4.6)	0.347	6 (3.2)	2 (2.0)	0.718	6 (2.5)	2 (3.7)	0.576
Gingivitis (gum bleed)	7 (2.4)	3 (1.3)	4 (9.1)	0.011	5 (2.6)	2 (2.0)	0.730	6 (2.5)	1 (1.9)	0.512
Melena (blood in stools)	5 (1.7)	2 (0.8)	3 (6.8)	0.026	2 (1.1)	3 (3.0)	0.345	5 (2.1)	0 (0.0)	0.279
Hematochezia (fresh blood in stools)	2 (0.7)	2 (0.8)	0 (0.0)	0.720	2 (1.1)	0 (0.0)	0.545	2 (0.8)	0 (0.0)	0.603
Hematemesis (vomiting blood)	11 (3.8)	10 (4)	1 (2.3)	0.484	5 (2.6)	6 (5.9)	0.199	9 (3.8)	2 (3.7)	0.666
Hematuria (blood in urine)	5 (1.7)	4 (1.6)	1 (2.3)	0.562	3 (1.6)	2 (2.0)	0.802	4 (1.7)	1 (1.9)	0.690
Petechiae (small spots on skin)	17 (5.8)	14 (5.6)	3 (6.8)	0.489	5 (2.6)	12 (11.9)	0.002	13 (5.5)	4 (7.4)	0.531
Purpura (reddish/bluish skin lesions)	2 (0.7)	2 (0.8)	0 (0.0)	0.720	1 (0.5)	1 (1.0)	0.648	2 (0.8)	0 (0.0)	0.663
Ecchymosis (larger reddish/bluish skin lesions)	4 (1.4)	4 (1.6)	0 (0.0)	0.517	2 (1.1)	2 (2.0)	0.612	3 (1.3)	1 (1.9)	0.562
**Clinical Signs**										
Abdominal tenderness	3 (1.0)	2 (0.8)	1 (2.3)	0.39	2 (1.1)	1 (1.0)	0.960	3 (1.3)	0 (0.0)	0.465
Hepatomegaly (liver enlargement)	24 (8.2)	18 (7.3)	6 (13.6)	0.134	19 (10.0)	5 (5.0)	0.18	20 (8.4)	4 (7.4)	0.340
Splenomegaly (spleen enlargement)	22 (7.6)	17 (6.8)	5 (11.4)	0.225	16 (8.4)	6 (5.9)	0.495	18 (7.6)	4 (7.4)	0.428
Pleural effusions	7 (2.4)	1 (0.4)	6 (13.6)	0.000	4 (2.1)	3 (3.0)	0.697	7 (2.9)	0 (0.0)	0.355
Ascites	7 (2.4)	3 (1.3)	4 (9.1)	0.011	6 (3.2)	1 (1.0)	0.428	7 (2.9)	0 (0.0)	0.355
**Management and Outcome**										
Duration of hospital stay [Mean (SD), days]	3.2 (1.9)	2.9 (1.7)	4.3 (2.7)	<0.001	3.1 (2.2)	3.4 (1.2)	<0.001	3.2 (1.9)	3.1 (1.9)	0.70
Fever from [Mean (SD), days]	6.37 (2.3)	6.5 (2.9)	5.9 (2.2)	0.174	6.32 (2.3)	6.5 (2.1)	0.921	6.4 (2.3)	6.5 (2.3)	0.884

**Abbreviations:** DENV = dengue virus; CHIKV = chikungunya virus; SD = standard deviation

*A p-value was calculated by the Chi-square test for categorical variables. Mann Whitney U test was used to calculate a p-value for quantitative variables. A p-value ≤0.05 was considered statistically significant.

Among a total of 291 dengue patients, 247 (84.9%) developed DF while 44 (15.1%) developed DHF. There was no significant difference (p-value>0.05) in the age and gender distribution among DF and DHF patients. However, clinical symptoms including myalgia/arthralgia, rash, anorexia, and periorbital puffiness were significantly more frequent (p-value<0.05) in DHF patients. Additionally, vomiting, retro-orbital pain, and periorbital pain were also more frequent although not significantly in DHF patients. In contrast, diarrhea, sore throat, cold/clammy skin, and restlessness were significantly more frequent (p-value<0.05) in DF patients. Among hemorrhagic manifestations, gingivitis and melena were significantly more frequent (p-value<0.05) in DHF patients. Similarly, pleural effusions and ascites were significantly more frequent in the DHF as compared to DF patients. Whereas, abdominal tenderness, hepatomegaly, and splenomegaly were also more frequent in DHF patients. The mean length of hospital stay was significantly increased in the case of DHF (4.3 days) as compared to DF (2.9 days) patients (Table 2).

Complete blood count revealed thrombocytopenia (platelets count <150,000 cells/mm^3^) in all 291 (100%) patients, while 237 (81.4%) patients reported with platelets count less than 100,000 cells/mm^3^ and a severe thrombocytopenia (platelets count <25000 cells/mm^3^) was reported in 39 (13.4%) patients. Low hemoglobin and erythrocytopenia were observed in 108 (37.1%) and 107 (36.8%) patients, respectively. Among a total of 291 patients, 91 (31.3%) were positive for DENV NS1 antigen, while 34 (11.7%) were positive for IgM and 16 (5.5) were positive for IgG antibodies only. Interestingly, various combinations of NS1 antigen and dengue antibodies were also detected in the patients which are summarized in Table 3.

**Table 3:** Laboratory findings of dengue patients reported from Lahore and Rawalpindi.

Laboratory findings	Frequency *n* (%)
***Hematological findings***	
Thrombocytopenia (<150,000) at presentation	291 (100)
<100,000	237 (81.4)
<50,000	93 (32)
<25,000	39 (13.4)
<10,000	4 (1.4)
Erythrocytopenia	107 (36.8)
Low hemoglobin	108 (37.1)
Leukopenia	134 (46)
HCT level on admission [median (SD)]	39.5 (5.8)
***Dengue immunodiagnosis (n = 291)***	
NS1 antigen	91 (31.3)
IgM	34 (11.7)
IgG	16 (5.5)
NS1+ IgM	67 (23)
NS1 + IgG	23 (7.9)
NS1 + IgM + IgG	30 (10.3)
IgM + IgG	30 (11.3)

**Abbreviations:** HCT = hematocrit; SD = standard deviation; NS1 = dengue virus non-structural antigen 1; IgM = immunoglobulin M; IgG = immunoglobulin G

**Fig 2:**
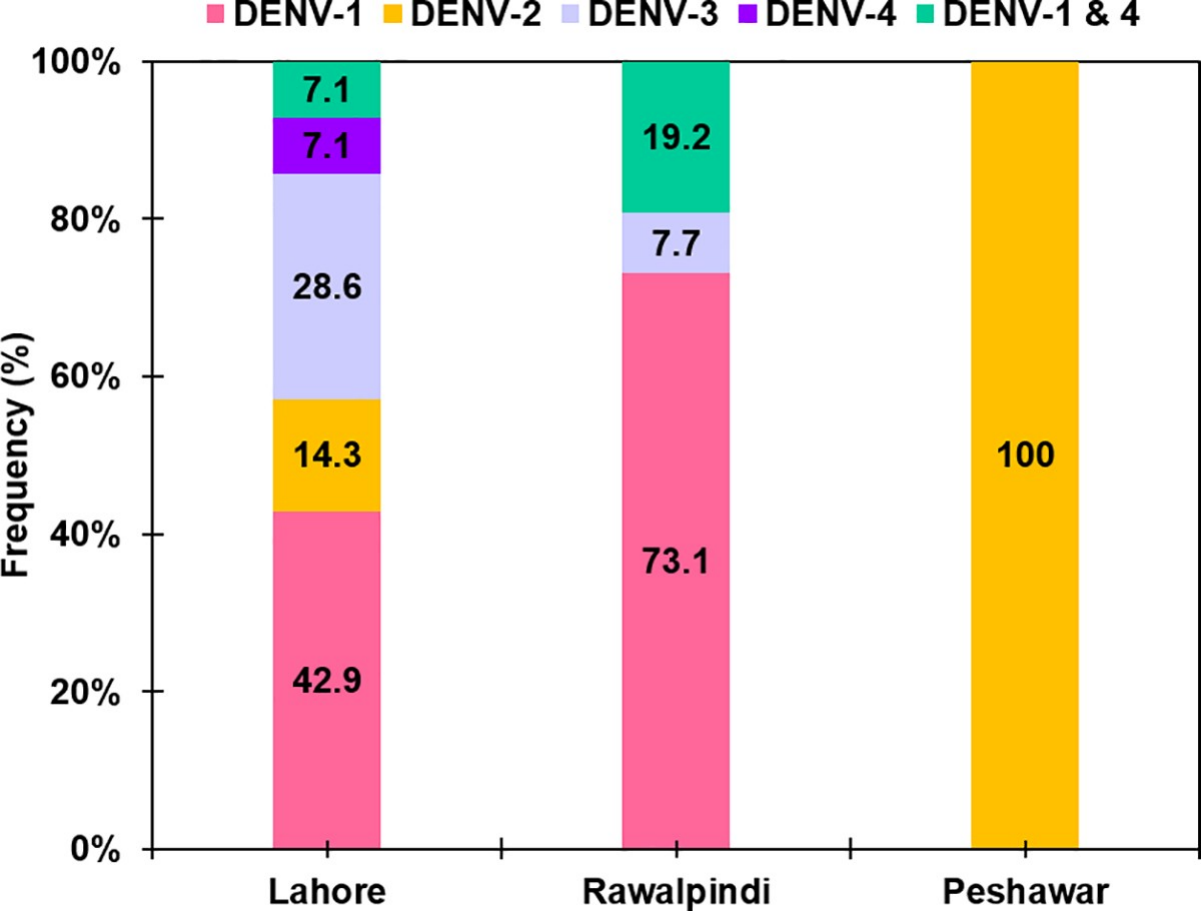
Prevalence of dengue virus serotypes circulating in different districts of Punjab and Khyber Pakhtunkhwa provinces of Pakistan in 2016–18. Among a total of 510 NS1 positive samples from dengue patients, 155 (30.4%) were PCR positive including 14 from Lahore, 26 from Rawalpindi, and 115 from Peshawar.

Quantitative reverse transcriptase PCR (qRT-PCR) was performed to determine the DENV serotypes circulating in Lahore and Rawalpindi. Among a total of 211 (72.5%) dengue patients (including 91 NS1 antigen, 67 NS1+IgM, 23 NS1+IgG, and 30 NS1+IgM+IgG positive samples), 40 (19%) were PCR positive. All four serotypes were detected in the patients’ sera and DENV-1 was the most commonly reported in 25 (62.5%) samples, followed by DENV-3 in 6 (15%), DENV-2 in 2 (5%), and DENV-4 was detected in only 1 (2.5%) sample. Interestingly, concurrent infections of DENV-1 + DENV-4 were detected in 6 (15%) samples (Table 4 and Fig 2).

**Table 4:** Prevalence of DENV serotypes in Lahore, Rawalpindi, and Peshawar districts in 2016–18.

Year	Districts	Total samples tested	NS1 positive *n* (%)	PCR positive* *n* (%)	Dengue serotypes				
					DENV-1	DENV-2	DENV-3	DENV-4	Mixed
***Hospital-based studies***									
2016	Lahore	25	14 (56)	9 (64.3)	6	1	0	1	1
2017	Lahore, Rawalpindi	152	101 (66.5)	16 (15.8)	8	0	5	0	3
2018	Lahore, Rawalpindi	114	96 (84.2)	15 (15.6)	11	1	1	0	2
	**Total**	**291**	**211 (72.5)**	**40 (19)**	**25**	**2**	**6**	**1**	**6**
***Community-based study***									
2017	Peshawar	6000	299 (4.9)	115 (38.5)	0	115	0	0	0
	**Grand Total**	**6291**	**510 (8.1)**	**155 (30.4)**	**25**	**117**	**6**	**1**	**6**

**Abbreviations:** NS1 = dengue virus non-structural antigen 1; PCR = polymerase chain reaction; DENV = dengue virus

*NS1 antigen, NS1 + IgM, NS1 + IgG, and NS1 + IgM + IgG positive samples were used for DENV serotyping using qRT-PCR. The PCR percentages were calculated based on the number of NS1 positives in the denominator.

### Prevalence of comorbidities

**Fig 3:**
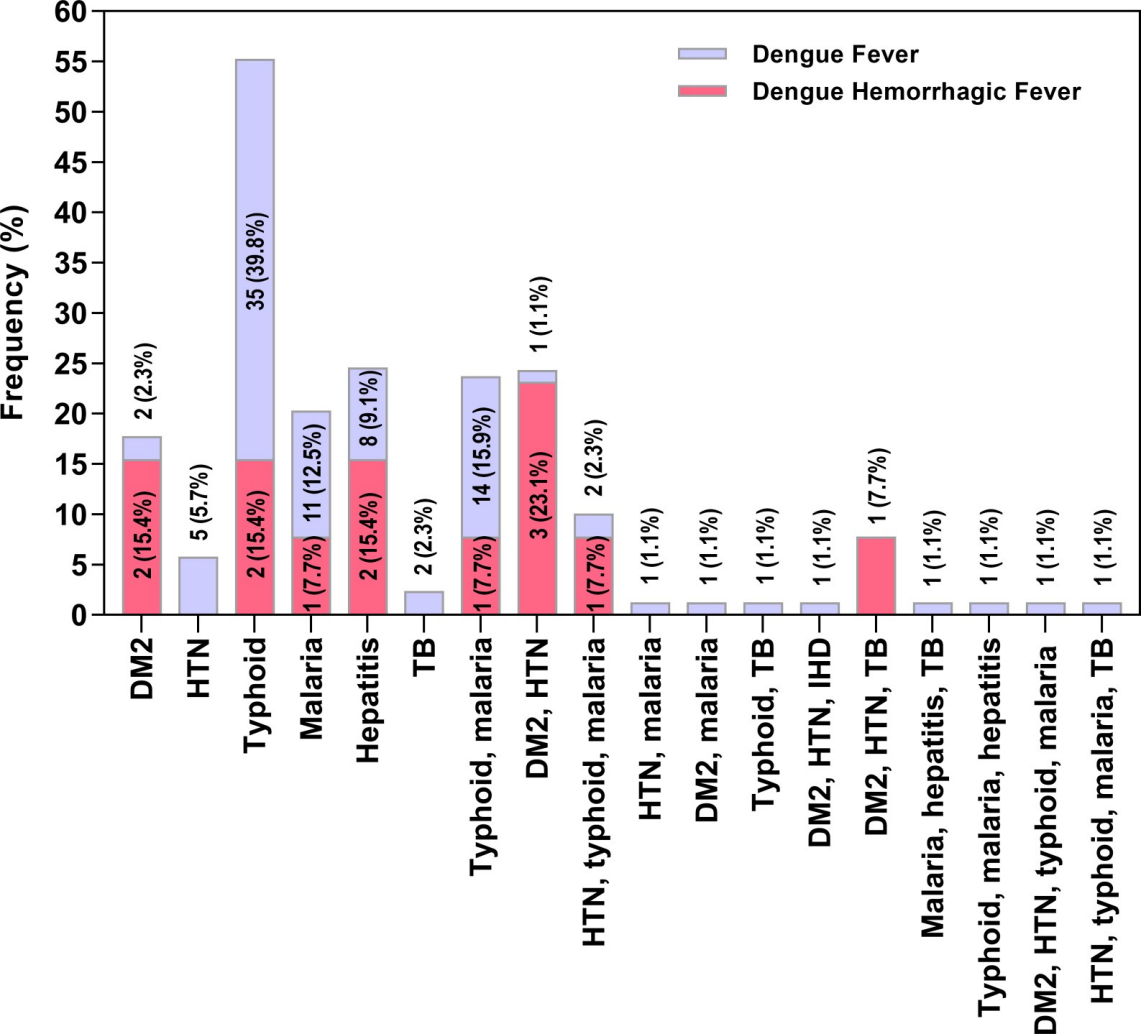
Prevalence of different comorbidities (*n* = 101) in patients reported with dengue fever (*n* = 88) and dengue hemorrhagic fever (*n* = 13). **Abbreviations:** DM2 = type 2 diabetes mellitus; HTN = hypertensions; IHD = ischemic heart disease; TB = tuberculosis.

A total of 101 (34.7%) patients were diagnosed with various comorbidities including 75 (74.3%) males and 26 (25.7%) females. Among them, 70 (69.3%) patients were diagnosed with only one type of comorbidity, while 31 (30.7%) were diagnosed with multiple comorbidities (Fig 3). Among non-communicable diseases (NCDs), type 2 diabetes mellitus (DM2) was diagnosed in 4 (4%) and hypertension (HTN) in 5 (5%) patients. Patients were also diagnosed with different co-infections most commonly including typhoid in 37 (36.6%), followed by malaria in 12 (11.9%), viral hepatitis (HBV/HCV) in 10 (9.9%), and tuberculosis (TB) in 2 (2%) patients (Fig 3).

The median age of the dengue patients with comorbidities was 33 years (range = 10 to 85 years), including 9 (8.9%) children (till 16 years), and 92 (91.1%) adults. Most of the patients including 36 (35.6%) belonged to the age group of 16 to30 years followed by 34 (33.7%) from the age group of 31 to 45 years. The least number of patients (8.9%) were reported from the age group of <15 years. Among clinical symptoms, headache, abdominal pain, diarrhea, myalgia/arthralgia, cough, sore throat, vomiting, cold skin/clammy skin, and restlessness were significantly more frequent (p-value<0.05) in patients reported with comorbidities than DF alone (Table 2). Among hemorrhagic manifestations, petechiae were most commonly associated with patients with comorbidities. However, no significant difference in the clinical signs among both groups was observed. The length of hospital stay was significantly (p-value<0.001) increased for patients reported with comorbidities (Table 2). Among total 44 patients reported with various comorbidities, 13 (29.6%) developed DHF, including 2 (15.4%) with DM2, 2 (15.4%) with typhoid, 1 (7.7%) with malaria, 2 (15.4%) with viral hepatitis, 1 (17.7%) with typhoid + malaria, 3 (23.1%) with DM2 + HTN, 1 (7.7%) with HTN + typhoid + malaria and 1 (7.7%) with DM2 + HTN + TB (Fig 3). Overall, DF was diagnosed in 6 (2.4%) and DHF in 6 (13.6%) patients with DM2 alone or in combinations with other diseases. The number of DHF cases was significantly more frequent (p-value = 0.004) in patients with DM2 as compared to DF alone (Fig 3). The odds ratio (OR) was calculated to determine the association of DM2 in developing DHF. The odds to develop DHF were ~2 times more frequent (OR 1.94, 95% CI 0.61 to 6.18, p-value = 0.004) in patients with DM2 than in DENV infection alone (Fig 3). Typhoid was diagnosed in the highest numbers, however, no relationship with the severity of the disease was found.

### Circulating DENV serotypes in Peshawar

A total of 6000 healthy participants were enrolled through walk-in camps and blood samples were collected from them as per standard protocols. The median age of the participants was 23 years (range = 2 to 80 years) and included 2772 (46.2%) males and 3238 (53.8%) females. Among a total of 6000 participants, 299 (4.98%) were found positive for the DENV NS1 antigen. These NS1 antigen-positive samples were further analyzed for DENV serotypes. DENV-2 was the only circulating serotype detected in the local population in 115 (38.5%) samples. Fig 2 compares DENV serotypes circulating in Lahore, Rawalpindi, and Peshawar.

### Chikungunya virus co-infection

**Fig 4:**
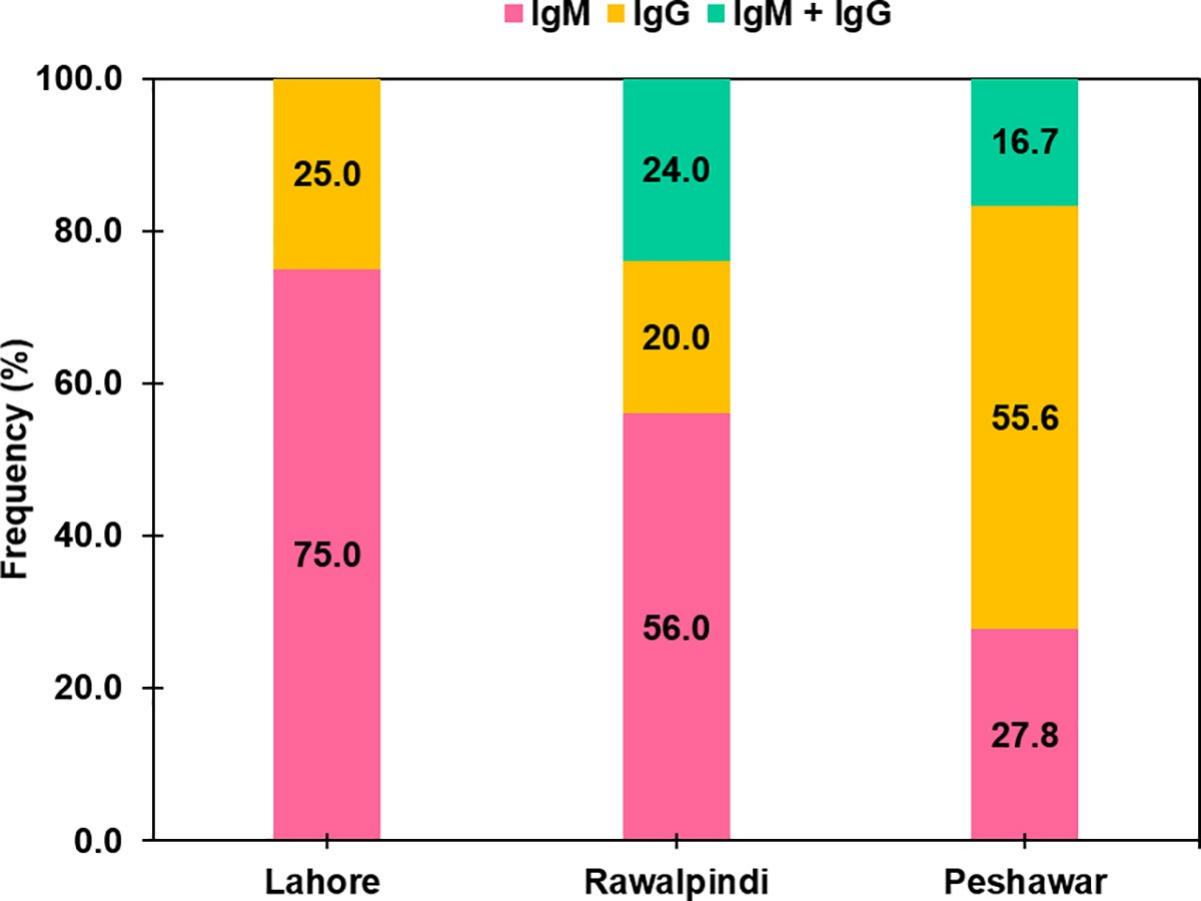
Seroprevalence of Chikungunya virus co-infection in dengue cases reported from Lahore (*n* = 4), Rawalpindi (*n* = 50) and Peshawar (*n* = 18).

Among a total of 590 dengue positive blood samples (291 from Lahore and Rawalpindi + 299 from Peshawar), 72 (12.2%) were positive for CHIKV infection. Their median age was 28 (range 7–68) years. ELISA showed that 36 (50%) were positive for CHIKV IgM, 21 (29.2%) for IgG, and 15 (20.2%) were positive for both IgM and IgG antibodies. Among them, CHIKV infection was detected in 54/291 (18.5%) hospitalized patients from Lahore and Rawalpindi, while 18/299 (6.02%) cases were detected from the community from Peshawar. Fig 4 shows the seroprevalence of CHIKV infection in three cities of Pakistan.

Comparison of the 54 hospitalized patients’ data showed that retro-orbital pain and restlessness were significantly more frequent (p-value = 0.004) in 20 (37.0%) and 18 (33.3%) patients, respectively with DENV-CHIKV co-infection as compared to DENV infection alone. However, no other significant differences (p-value >0.05) in demographic features, signs, and symptoms, and clinical outcome were noted among both groups (Table 2).

Data were also analyzed for any association of DENV-CHIKV coinfection and other co-morbidities in the development of severe dengue fever. Among 54 (18.5%) patients reported with DENV-CHIKV coinfection, 4 (7.4%) were reported with type DM2 and 5 (9.3%) with hypertension. Similarly, other coinfections including malaria was detected in 10 (18.5%), viral hepatitis in 3 (5.6%), typhoid in 10 (18.5%), and TB in 1 (1.9%) patient. Among a total of 54 DENV-CHIKV patients, 3 (5.6%) developed DHF including only 1 (33.3%) patient co-infected with viral hepatitis.

## Discussion

Dengue hemorrhagic fever/dengue shock syndrome (DHF/DSS) is the most serious manifestation of dengue fever characterized by thrombocytopenia, hemorrhagic manifestations, vascular leakage, hypotension, and shock which might lead to organ failure and death [24]. Epidemiological studies suggest that primary dengue infections are generally asymptomatic but they infrequently manifest as a more severe form of the disease [30,31]. For more than seven decades it was believed that primary infection with one of the serotypes confers lifelong immunity against homotypic DENV reinfection, however, few recent studies challenged the current understanding of DENV immunity. Forshey et al. [32] and Waggoner et al. [33] reported that homotypic primary DENV infection might not provide sterilizing immunity against specific serotypes and reinfections are possible. This finding will have implications for the modeling of DENV transmission as well as for vaccine development. Secondary infection (especially after a long time interval between the primary and secondary infection) with the heterotypic serotype might results in a more severe outcome through a less understood phenomenon of antibody-dependent enhancement (ADE) [4,34,35]. Therefore, the circulation of all four serotypes in major metropolitans of Pakistan is an alarming situation that could result in an outbreak of severe dengue fever in the subsequent years due to exposure to heterotypic serotype than the previous one.

DENV-1 was the predominant serotype detected from hospitalized patients in Lahore and Rawalpindi while DENV-2 was the only serotype detected from Peshawar where a large outbreak was reported for the first time in history in 2017. Peshawar city being the provincial capital of Khyber Pakhtunkhwa and the center for various socio-economic activities is an attractive place for the business communities and visitors who could contribute significantly to importing new DENV serotypes to the local population. Recently, mobile phone-based mobility data of ~40 million people from dengue-endemic areas in Pakistan showed that international/local travelers played a significant role in the spread of dengue infection to metropolitan cities like Peshawar in 2011 [36]. Therefore, it is not far away when all four serotypes will be circulating in the city. Therefore, the population of Peshawar is at a much higher risk of developing severe disease in the near future due to the enormous possibility of the introduction of another serotype(s) through travelers coming from other neighboring cities and abroad.

It is noteworthy that DENV-2 or DENV-3 remained the predominant DENV serotypes in Pakistan while the cases of DENV-1 and DENV-4 remained at low levels previously [14,16,18,20,21,37,38]. One study from Lahore also reported the predominance of DENV-4 during 2008 [17]. However, the current study observed the predominance of DENV-1 in samples collected from Lahore (42.9%) and Rawalpindi (73.1%) which is a recent phenomenon and observed for the first time (Fig 2). Co-circulation of multiple serotypes is itself an alarming situation but this change in the pattern of circulating DENV serotypes might result in a more serious outcome of dengue infections in the future due to exposure of population previously sensitized to DENV-2 and DENV-3. Similarly, Lardo et al. [39] reported an increase in the frequency of DHF cases with the re-emergence of DENV-3 in Kolkata, India.

Moreover, concurrent dengue infections (i.e. infection with multiple DENV serotypes) gained attention due to their association with severe dengue fever [39,40]. This study also detected a total of 6 cases of concurrent infection with DENV-1 and -DENV-4 from Lahore (*n* = 1) and Rawalpindi (*n =* 5) (Fig 2). On the contrary, none of the patients developed severe dengue fever however, follow-up studies instead of cross-sectional studies are recommended to determine the true outcome of such infections.

The outcome of DENV infection depends upon multiple intrinsic (host/physiological) and extrinsic (viral) factors. Similarly, comorbidities in dengue fever are considered to be a risk factor for the development of severe disease [41–43]. Various studies demonstrated previously that chronic comorbidities like diabetes, hypertension, allergies, and obesity were associated with severe manifestations of dengue fever [44–46]. It is also noteworthy that NCDs are becoming increasingly common in Asia especially in Southeast Asia where Pakistan also exists. The current study noted that DM2 contributed to the severity of the DENV infection. The odds to develop DHF were significantly more frequent (OR 1.94, 95% CI 0.61 to 6.18, p-value = 0.004) in patients with DM2 than in DENV infection alone.

Another important factor that might have contributed to the exacerbation of the disease included the age of the patients. The dengue generally affects children and young adults especially in the age group of 5 to 30 years [47], however, an increase in the severity of the disease (especially with comorbidities) has been seen in the older population around the globe [48,49]. Similarly, the patients diagnosed with comorbidities in this study were considerably older (p-value = 0.005) than the patients reported with dengue alone (Table 2). Further, the DHF patients with comorbidities were significantly older (median age = 38 years) than those without comorbidities (median age = 29 years, p-value <0.05).

In addition to contributing to the severity of the disease, comorbidities also contribute towards prolonged hospital stay thus contributing substantially towards an increase in the morbidity, and public health burden in the dengue-endemic countries [41–43]. The current study demonstrated an increase in the duration of hospital stay in patients with comorbidities. Therefore, comorbidities are not only associated with heightened morbidity but also inflict an economic burden on the healthcare system.

Among a total of 590 DENV positive blood samples, 72 (12.2%) were also positive for CHIKV infection which indicates co-circulation of viral infections in the community. CHIKV is an enveloped (70nm) single-stranded linear RNA (11.8Kb) alphavirus belonging to the family Togaviridae [50]. The symptoms of CHIKV infection are similar to DENV infection with fever and joint pain are the commonest symptoms. The CHIKV is transmitted by the same Aedes mosquitos as of DENV [51]. Le Coupanec et al. [52] demonstrated co-viral infections of CHIKV and DENV-2 after 4 and 5 days post-exposure respectively in the midgut and after 4 and 10 days post-exposure respectively in the salivary glands of *Ae aegypti* mosquitoes. Thus the co-infection might be carried out by consecutive bites of two female mosquitoes each carrying a different virus or by the bite of a single female mosquito co-infected with both viruses. Previously *Ae*. *albopictus* mosquitoes were collected in the vicinity of human cases co-infected with CHIKV and DENV [53]. The earliest records of CHIKV infections from Pakistan can be traced back to the 1980s [54], however, no epidemic had been recorded for the last three decades until recently in 2016 when a large number of cases of “mysterious” disease started to rise in the port city of Karachi (Sindh) which were later on diagnosed as CHIKV infections [55]. The disease spread rapidly to the other three provinces and reached the federal capital Islamabad by mid-2017 [56]. It is possible that the disease might already be circulating in the country but vastly remained unnoticed due to the lack of expertise, unavailability of diagnostic facilities, and lower number of cases reported previously.

## Conclusions

The co-circulation of all four DENV serotypes in the major metropolitan cities (Lahore and Rawalpindi) of Punjab is a worrisome situation as it could result in an outbreak of DHF/DSS in the future. DENV-2 was the only serotype detected from the Peshawar population and there is a high possibility of importation of other serotypes from dengue-endemic cities of Pakistan to Peshawar. Thus the population of Peshawar is at much higher risk of developing DHF/DSS due to secondary infections with heterologous DENV serotypes. Comorbidities (especially type 2 diabetes mellitus) play a significant role in the exacerbation of the disease by contributing to the severity of the disease. Further, comorbidities result in increased duration of hospital stay thus inflict an economic burden on the health system and the patients. The current study also detected DENV-CHIKV co-infections from Lahore, Rawalpindi, and Peshawar. The co-circulation of another arboviral disease in Pakistan will not only result in increased morbidity and mortality but will also inflict an economic burden on the already fragile health system.
